# Shortcomings of silhouette in single-cell integration benchmarking

**DOI:** 10.1038/s41587-025-02743-4

**Published:** 2025-07-30

**Authors:** Pia Rautenstrauch, Uwe Ohler

**Affiliations:** 1 Max-Delbrück-Center for Molecular Medicine in the Helmholtz Association (MDC), Berlin Institute for Medical Systems Biology (BIMSB), Berlin, Germany; 2 Humboldt-Universität zu Berlin, Department of Computer Science, Berlin, Germany; 3 Humboldt-Universität zu Berlin, Department of Biology, Berlin, Germany

**Keywords:** Data integration, Statistical methods, Computer science, Bioinformatics, Standards

## Abstract

Single-cell studies rely on advanced integration methods for complex datasets affected by batch effects from technical factors alongside meaningful biological variation. Silhouette is an established metric for assessing unsupervised clustering results, comparing within-cluster cohesion to between-cluster separation. However, silhouette’s assumptions are typically violated in single-cell data integration scenarios. We demonstrate that silhouette-based metrics cannot reliably assess batch effect removal or biological signal conservation and propose more robust evaluation strategies.

## Abstract (web summary)

Silhouette score is unsuitable as a metric for single-cell data integration.

## Main

Integrating single-cell data remains a key challenge because of increasing dataset complexity and volume. These datasets comprise batch effects arising from technical factors (for example, assays and protocol), alongside meaningful biological variation (for example, distinct tissue sampling regions), requiring rigorous evaluation of integration methods to ensure accurate integration and interpretation. We focus on methods for horizontal integration (a term coined by Argelaguet et al.^1^), defined as integrating datasets using shared features (for example, genes) aiming to remove batch effects while preserving biological variation. Although relevant to distinct output types, we focus on integrated embeddings, low-dimensional data representations derived from integration methods.

**Extended Data Fig. 1:**
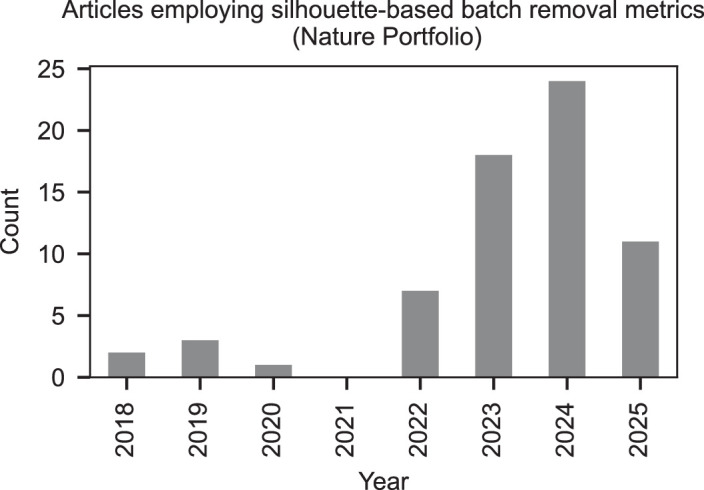
Usage of silhouette-based batch removal metrics in Nature Portfolio journals. Number of articles using silhouette-based batch removal metrics published in Nature Portfolio journals (last updated on April 10th, 2025).

Silhouette-based evaluation metrics, which we find are unreliable for horizontal integration, have become widely adopted to address this challenge. The metric ‘silhouette’ scores clustering quality by comparing within-cluster cohesion to between-cluster separation^2^ and was developed for evaluating unsupervised clustering results of unlabeled data (internal evaluation). In line with its original intent, silhouette was taken up for determining the optimal number of clusters in single-cell datasets for a given embedding^3,4^. More recently, silhouette has been adapted for evaluating horizontal data integration, for instance, to score bio-conservation by assessing how well cell type annotations (based on labeled data; that is, external evaluation) from distinct batches cocluster in distinct embeddings^5–7^. From 2017 onward, silhouette-based metrics have also been used for scoring batch effect removal^5,7–9^. Here, researchers attempt to invert the silhouette concept to score how well cells from distinct batches (external labels) mix. Silhouette-based metrics for both bio-conservation and batch removal have been widely adopted across the field, as evidenced by their application in multiple large-scale benchmarks^10–12^. In Nature Portfolio journals alone, we found evidence for their use in 66 publications for evaluating batch removal (Extended Data Fig. 1 and Supplementary Table 1). Notably, these studies extend beyond single-cell sequencing data, encompassing spatial transcriptomics and image-based single-cell modalities.

Silhouette-based metrics suffer from fundamental, largely overlooked limitations for evaluating horizontal data integration. To expose these issues, we first formalize the silhouette score and its adaptations for single-cell integration tasks. Using simple simulations, we demonstrate how the metric’s assumptions are violated under basic conditions, misleadingly rewarding poor integration. We then validate these findings in real-world datasets, proving that these issues persist beyond theoretical scenarios.

The silhouette coefficient for a cell *i* assigned to a cluster ${C}_{k}$, denoted ${s}_{i}$, is defined as follows. Given ${a}_{i}$ (the mean distance between a cell *i* and all other cells in the same cluster ${C}_{k}$) and ${b}_{i}$ (the mean distance between a cell *i* and all other cells in the nearest (neighboring) other cluster ${C}_{l}$, where $l\ne k$), ${s}_{i}$ is given by

1 $${s}_{i}=\frac{{b}_{i}-{a}_{i}}{\max ({a}_{i},{b}_{i})}$$

Conventionally and if not stated otherwise, Euclidean distance is used. Note that ${s}_{i}$ is only defined for ${2} \le n \;{\rm{clusters}} \le n \;{\rm{cells}}-1$ and ranges between −1 and 1, with 1 indicating good cluster separation (${a}_{i}\ll {b}_{i}$), values near 0 indicating cluster overlap (${a}_{i}={b}_{i}$) and −1 indicating wrong cluster assignment (${a}_{i}\gg {b}_{i}$). In contrast to the use of silhouette for internal clustering evaluation (unsupervised clustering), for scoring data integration in the single-cell field, cells are not assigned to clusters in a data-driven manner, for example, by the result of a clustering algorithm, but by external information, such as cell type or batch labels.

For scoring bio-conservation, cell type labels serve as cluster assignments. First, the average silhouette width (ASW) is calculated across all cells (unscaled cell type ASW). Following common practice, we use a rescaled version:

2 $${\rm{Cell}}\;{\rm{type}}\;{\rm{ASW}}=({\rm{unscaled}}\;{\rm{cell}}\;{\rm{type}}\;{\rm{ASW}}+1)/2$$

Notably, a score of 0.5 corresponds to an unscaled ASW of 0, indicating overlaps between cell types, an undesirable outcome. Higher values indicate better performance.

For scoring batch effect removal, batch labels serve as cluster assignments. Here, the goal is to measure cluster overlap rather than separation. Considering this context, researchers made the assumption that silhouette values ${s}_{i}$ around 0 indicate a high level of batch overlap. Two approaches exist.

Early adoptions, which remain in use, use a simple formulation where all cells from a given batch are assigned to a single cluster, which we refer to as batch ASW (global). This approach often computes 1 − batch ASW (global) or 1 − |batch ASW (global)|, with higher scores interpreted as better performance.

Luecken et al.^11^ acknowledged problems with differences in cell type composition between batches and thus introduced a modified version of batch ASW computed separately for each cell type. For a given cell type label $j$ with $|{C}_{j}|$ cells, the score is calculated as:

3 $${\rm{Batch}}\;{\rm{AS}}{{\rm{W}}}_{{j}}\;({\rm{cell}}\;{\rm{type}})=\frac{1}{|{C}_{j}|}\sum _{{i}\epsilon \,{C}_{j}}1-|{s}_{i}|$$

The final batch ASW (cell type) score (batch ASW from here on) is obtained by averaging across the scores for all cell type labels.

**Fig. 1:**
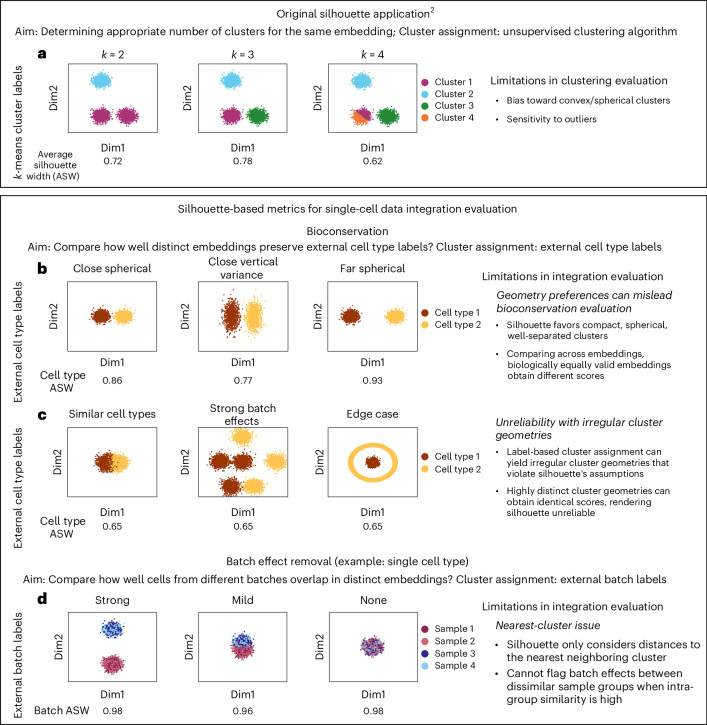
Silhouette’s assumptions are not met in data integration contexts. **a**, Silhouette was designed to select a suitable cluster number for a single embedding, with cluster membership resulting from unsupervised algorithms^2^. **b**–**d**, In data integration, we compare distinct embeddings and assign cluster membership by external labels: cell type (**b**,**c**) or batch (**d**). **b**, Silhouette’s bias for compact, spherical clusters does not reflect integration quality. **c**, Label-based clusters can have irregular shapes, violating silhouette’s assumptions and yielding unreliable scores. **d**, Silhouette’s focus on nearest neighboring clusters misses remaining batch effects if samples are partially integrated, limiting its sensitivity. All data shown are 2D simulated examples.

When repurposing the silhouette metric for evaluating horizontal data integration, researchers make two key changes compared to its original application. First, they use label-based rather than algorithmic cluster assignment. Second, they compare silhouette scores across the outputs of different methods (across embeddings) instead of relative to the output of a single method. We demonstrate how these and other conceptual changes inherently constrain the silhouette metric’s effectiveness for assessing horizontal integration using two-dimensional (2D) simulated data (Fig. 1).

Concerning bio-conservation evaluation, when comparing silhouette scores across distinct methods’ outputs, silhouette’s inherent preference for compact, spherical, well-separated clusters conflicts with biological reality, where such geometric properties bear no meaningful relationship to cellular state. This manifests in the metric resulting in different scores for distinct but biologically equally valid embeddings (Fig. 1b). Additionally, label-based assignments can produce irregular cluster geometries that would never emerge from algorithmic clustering (for example, batch-induced distortions), violating the metric’s assumption about cluster shapes. Silhouette’s behavior becomes unreliable, as demonstrated by identical silhouette scores representing radically different scenarios (Fig. 1c).

**Extended Data Fig. 2:**
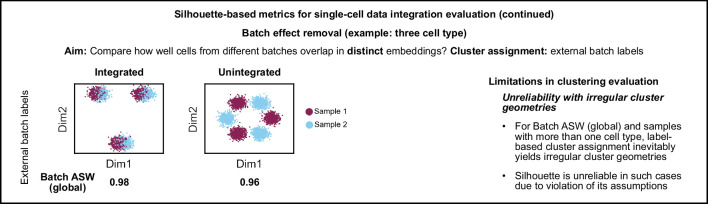
Silhouette’s assumptions are not met in data integration contexts (continued). In data integration, we compare distinct embeddings and assign cluster membership by external labels - here: batch. Label-based clusters can have irregular shapes, violating silhouette’s assumptions and yielding unreliable scores. This issue is particularly evident with Batch ASW (global) when more than one cell type is present.

Concerning batch effect removal, irregular cluster geometries are the default for batch ASW (global), where all cells from a given batch are forced into a single cluster regardless of cell type diversity, producing erratic scores that fail to reflect integration quality (Extended Data Fig. 2), which is why we generally discourage its use. Additionally, silhouette (Eq. (1)) considering the mean distance between a cell *i* and all other cells in the nearest (neighboring) other cluster ${C}_{l}$ (${b}_{i}$) is problematic in the batch removal context, affecting both batch ASW (global) and the cell type-adjusted batch ASW. For simplicity, consider integrating multiple datasets (samples) with a single cell type, where the aim is to score cluster overlap and not separation. A value for ${s}_{{i}}$ around 0 is attainable if a given cluster overlaps with just a single other cluster and could still be very distinct from all other remaining ones. Thus, silhouette-based batch removal metrics can result in maximal scores when all samples are integrated with subsets of the other samples despite remaining strong batch effects (Fig. 1d), which we call ‘nearest-cluster issue’.

**Fig. 2:**
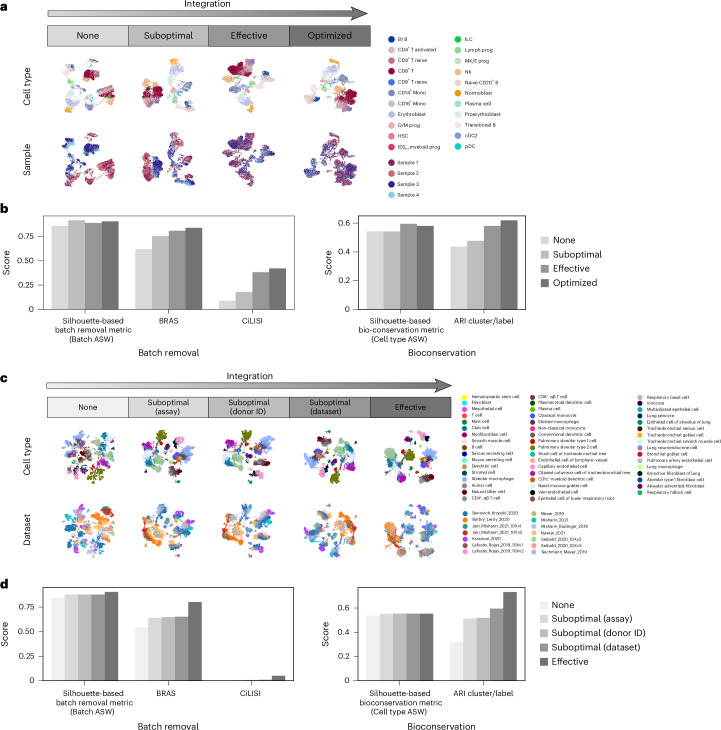
Silhouette-based metrics are unreliable for assessing bio-conservation and batch effect removal. **a**, Uniform manifold approximation and projections (UMAPs) of NeurIPS minimal example embeddings integrated with increasing success, colored by cell type and sample. **b**,**d**, Batch removal metrics: batch ASW, BRAS and an alternative cell-type-adjusted diversity score, CiLISI. Bio-conservation metrics: cell type ASW and ARI. **c**, UMAPs of healthy HLCA embeddings integrated with increasing success colored by cell type and dataset, shown for a consistent random 10% data subset. Suboptimal embeddings were obtained through batch-aware HVG selection for specified batch variables.

**Extended Data Fig. 3:**
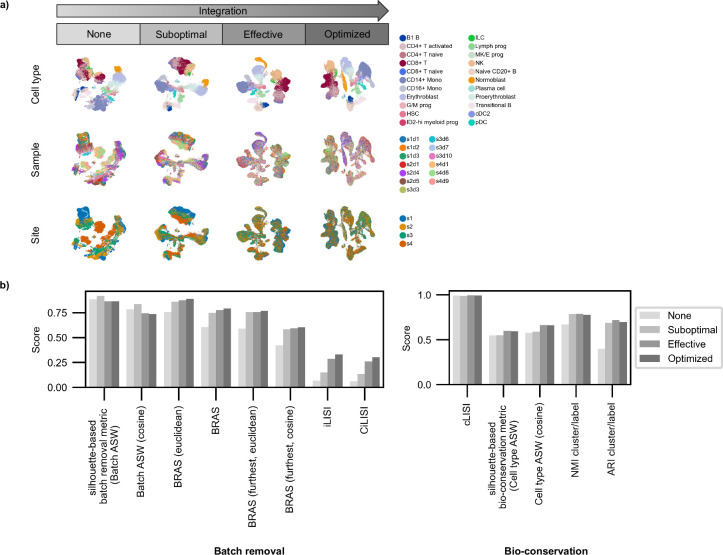
Silhouette-based metrics are unreliable for assessing bio-conservation and batch effect removal (full NeurIPS data). (**a**) UMAPs of full NeurIPS data embeddings integrated with increasing success, colored by cell type, sample, and site. (**b**) Extended evaluation metrics.

These limitations are also painfully obvious in real datasets. For simplicity, we limit our analyses to healthy samples and treat interdonor variation as negligible noise. A discussion of strategies for evaluating heterogeneous sample integration can be found in Supplementary Note 2. We discovered the nearest-cluster issue for batch ASW in the context of the NeurIPS 2021 challenge^13^, where the benchmark data have a nested experimental design and intersite technical variation is larger than intrasite variation between samples of distinct donors. Choosing a single-cell RNA sequencing (scRNA-seq) subset (‘minimal example’) of this data with four batches nested into two groups (sites), we compare metric performance on unintegrated, suboptimally integrated and effectively integrated and optimized (with respect to batch removal) integrated data with liam^14^ (Fig. 2a). Batch ASW fails to rank embeddings accurately and even favors worse embeddings with stronger batch effects (Fig. 2b), with the same observations applying to the full dataset (Extended Data Fig. 3b). Cell type ASW assigns almost identical scores to unintegrated and suboptimally integrated embeddings of the minimal example and the full data (Fig. 2b and Extended Data Fig. 3b), reflecting fundamental limitations in its discriminative power.

**Extended Data Fig. 4:**
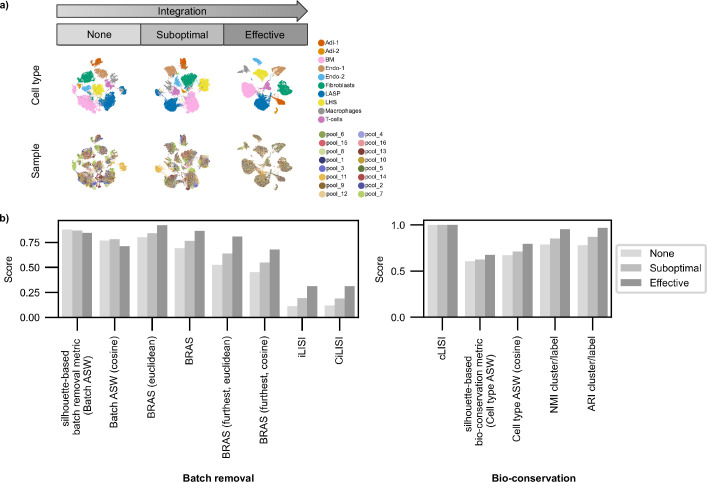
Silhouette-based metrics are unreliable for assessing bio-conservation and batch effect removal (HBCA data). (**a**) UMAPs of HBCA embeddings integrated with increasing success, colored by cell type and sample. (**b**) Extended evaluation metrics.

The violation of silhouette’s assumptions and resulting unreliability is not limited to datasets with controlled nested experimental designs. We demonstrate this by extending our analysis to two recent atlas-level studies, which differ in batch effect severity, cell type complexity and granularity of provided annotations: the healthy subset of the Human Lung Cell Atlas (HLCA)^15^ and the genetically diverse Human Breast Cell Atlas (HBCA)^16^. Using author-provided integrated embeddings, we compare those to unintegrated and naively integrated embeddings (Fig. 2c and Extended Data Fig. 4a). For HLCA, the batch ASW metric shows limited discriminative power but ranks embeddings correctly (Fig. 2d), whereas, for HBCA, it inversely ranks embeddings, favoring the worst integration (Extended Data Fig. 4b). Regarding bio-conservation, cell type ASW indicates comparable performance for naive and integrated embeddings in HLCA (Fig. 2d). However, in HBCA, which has well-separated cell types and limited batch effects, cell type ASW retrieves the expected ranking (Extended Data Fig. 4b).

**Extended Data Fig. 5:**
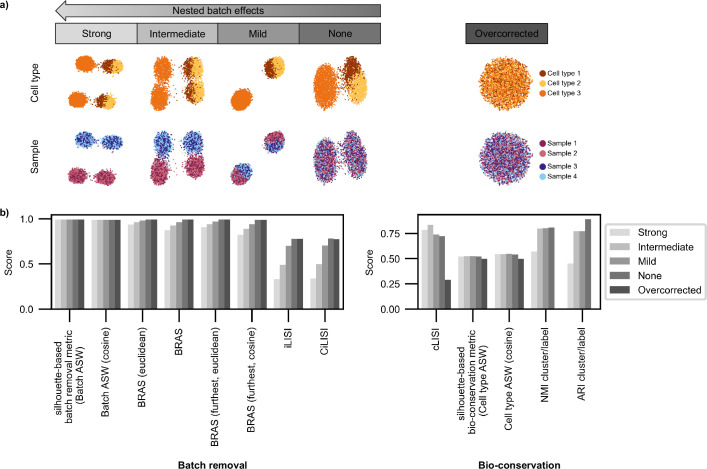
Silhouette-based metrics are unreliable for assessing bio-conservation and batch effect removal (simulated scRNA-seq data). (**a**) UMAPs of embeddings of simulated scRNA-seq data with nested batch effects between groups of samples with decreasing levels of batch effects between groups, colored by cell type and sample. (**b**) Extended evaluation metrics.

**Extended Data Fig. 6:**
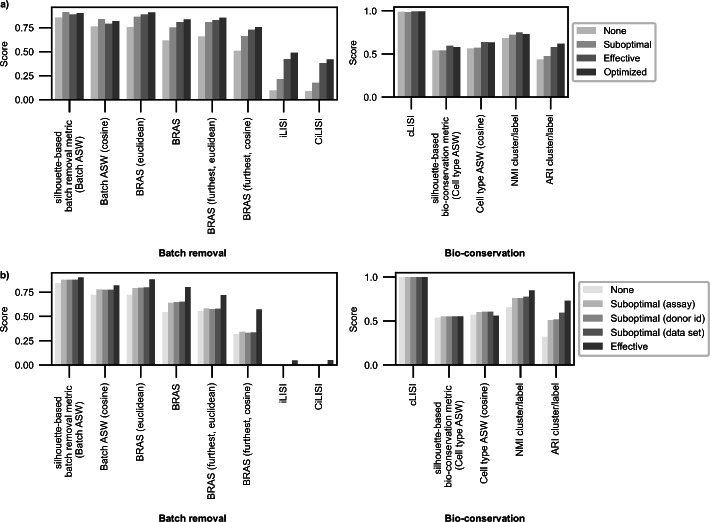
Silhouette-based metrics are unreliable for assessing bio-conservation and batch effect removal (NeurIPS data minimal example and HLCA data). Extended evaluation metrics. Batch removal and bio-conservation metrics (**a**) for NeurIPS real data minimal example and (**b**) HLCA (cf. Figure 2).

Single-cell integration benchmarking is an area of active research, which has seen large-scale coordinated efforts and typically includes a multitude of metrics extending beyond silhouette-based metrics^10–12,17,18^. Unanimously, it has been suggested that two classes of metrics should be considered to score horizontal data integration: batch removal and bio-conservation metrics^10,11,18^, which we introduce in detail in Supplementary Note 1. Concerning alternatives to silhouette for evaluating batch effect removal robust to the nearest-cluster issue, we find that combining a cell-type-adjusted local mixing batch removal with bio-conservation metrics on a cell type level is a successful strategy. For example, applying CiLISI (cell type integration local inverse Simpson’s index)^19^ with adjusted Rand index (ARI) leads to accurate rankings across datasets with the bio-conservation metric flagging overcorrection (Extended Data Fig. 5). It is also possible to ‘fix’ the silhouette-based metric batch ASW to be robust to the nearest-cluster issue by redefining ${b}_{i}$ as the mean distance between a cell *i* and all other cells in any other cluster ${C}_{l}$ with $l\ne k$. Changing Euclidean to cosine distance results in higher discriminative power. We call this metric batch-removal-adapted silhouette (BRAS; available through the scib-metrics package as of version 0.5.5; further details in Extended Data Figs. 3–6 and Methods, including a BRAS variant considering the furthest other cluster). Like CiLISI, the BRAS metric also accurately ranks all real and simulated scRNA-seq data (Fig. 2b,d and Extended Data Figs. 3b–5b and 6). The notable BRAS–CiLISI score divergence in HLCA embeddings (Fig. 2d) reflects their distinct focuses; while CiLISI evaluates (cell-type-adjusted) local batch mixing, BRAS is less sensitive to local compositional differences. Metric selection and weighting should align with integration objectives, as discussed in Supplementary Note 2; a discussion on how other identified silhouette limitations affect BRAS is provided in Supplementary Note 4. In search for alternatives to the unreliable silhouette for evaluating bio-conservation at the cell type annotation level, cLISI exhibits low discriminative power. However, the external clustering metrics ARI and normalized mutual information (NMI) reliably rank embeddings as anticipated (Fig. 2b,d and Extended Data Figs. 3b–5b and 6). Details on how clustering strategies influence ARI and NMI can be found in Supplementary Note 3; additional metrics scoring other aspects of horizontal integration are presented in Supplementary Note 1.

Our investigation reveals the inadequacy of currently prevalent silhouette-based evaluation metrics for assessing data integration caused by the violation of silhouette’s underlying assumptions. Silhouette’s inability to handle biologically realistic, nonconvex clusters persists across bio-conservation and batch removal evaluation, with the nearest-cluster issue further compounding batch removal evaluation. We outline robust alternatives, including a batch removal metric adjusting silhouette to be more robust to the discussed limitations, and urge discontinuing unadjusted silhouette-based metrics in data integration benchmarking. This is required to ensure reliable method assessment and method choice impacts downstream analyses.

## Methods

### Data

#### Simulated data (2D)

We generated datasets using scikit-learn’s make_blobs (Gaussian clusters, version 1.5.2)^20^ and custom geometric patterns to demonstrate silhouette’s original application and its limitations for evaluating horizontal data integration (Fig. 1).

For unsupervised clustering assessment, silhouette’s original application, we simulated three true clusters and applied *k*-means clustering (*k* = 2, 3 or 4) to produce distinct cluster assignments (dataset size: 4,000 data points each).

To demonstrate limitations in the bio-conservation context, we generated well-separated clusters with varying intercluster distances and variances (dataset size: 2,000 data points each) and shape-distorted datasets mimicking batch effects (including an edge case; 6,000 data points each).

To address limitations in the batch removal context, we generated two datasets (integrated and unintegrated) with three cell types across two samples to demonstrate the distance and shape sensitivity of batch ASW (global). Additionally, we modeled increasing vertical offsets between groups of similar samples for a single cell type to demonstrate nearest-cluster limitations (dataset size: 4,000 data points each).

The simulated datasets include simulated sample (batch variable) and cell type annotations. Parameters are detailed in Simulate_2D_data.ipynb.

#### Real data (NeurIPS dataset)

We used a benchmarking dataset from the NeurIPS 2021 Multimodal Single-Cell Data Integration competition, specifically designed to contain nested batch effects for evaluating integration. In particular, Luecken et al.^17^ profiled bone marrow mononuclear cells from multiple donors across distinct sites, with intersite batch effects being larger than intrasite batch effects between samples from distinct donors. For demonstration purposes, we only use the scRNA-seq data of the Multiome data accessible through the Gene Expression Omnibus (GEO; GSE194122), specifically a preprocessed AnnData object provided as a supplementary file. We further used a minimal data subset (minimal example) to illustrate the unreliable behavior of silhouette-based metrics with nested batch effects with four samples from four donors from two distinct sites (s1d1, s1d3, s4d8 and s4d9) comprising 24,704 cells for our main figure panels (Fig. 2a,b), which we renamed to samples 1, 2, 3 and 4, respectively. We also consider the full dataset comprising 69,249 cells, with results shown in Extended Data Fig. 3. The author-provided metainformation ‘batch’ and ‘cell_type’ were used as the batch (labeled ‘sample’ in figures) and cell type variable in our analyses.

#### Real data (HLCA)

The core integrated HLCA^15^ was used, comprising 584,944 healthy cells from five assays spanning 14 datasets and 107 donors. The data were accessed through CellxGene (‘An integrated cell atlas of the human lung in health and disease (core)’; https://datasets.cellxgene.cziscience.com/b351804c-293e-4aeb-9c4c-043db67f4540.h5ad). The author-provided metainformation ‘dataset’ and ‘cell_type’ were used as the batch (labeled ‘dataset’ in figures) and cell type variable in our analyses.

#### Real data (HBCA)

We used HBCA^16^, comprising 51,367 healthy cells from one assay and 82 donors that were processed in 16 pools, referred to as ‘donor_id’, which presents the most fine-grained annotation for sample origin available. The data were accessed through CellxGene (‘snRNA-seq analyses of breast tissues of healthy women of diverse genetic ancestry’; https://datasets.cellxgene.cziscience.com/63a485bc-cac7-49d2-83ed-8e07ca4efa2a.h5ad). The author-provided metainformation ‘donor_id’ and ‘author_cell_type’ were used as the batch (labeled ‘sample’ in figures) and cell type variable in our analyses.

#### Simulated data (scRNA-seq)

Drawing inspiration from Andreatta et al.^19^ and a recommendation of the Splatter developer (https://github.com/Oshlack/splatter/issues/99; last accessed April 10, 2025), we simulate five scenarios with decreasing levels of nested batch effects with the Splatter package^21^ (version 1.26.0). Each scenario was composed of four samples (used as batch variable in our analyses) with three cell types nested in two groups, meaning that the samples within a group were more similar to each other than between the groups. The scenarios were ‘strong’, ‘intermediate’, ‘mild’, ‘none’ (with no nested batch effects) and ‘overcorrected’ (with neither nested batch effects nor biological cell type signal). We first simulated data with two samples of 2,000 cells stemming from three distinct cell types with varying proportions. We varied the nested batch effect for the different scenarios using the batch.facLoc and batch.facScale parameters. We then selected half of the cells of the two samples and added small noise factors to them, resulting in four samples nested into two groups of 1,000 cells each, with the total datasets comprising 4,000 cells each. The noise factor stemmed from another simulated data matrix without batch and cell type structure where we used a small library size parameter lib.scale. In the overcorrected scenario, we chose no differential expression between cell types and samples.

### Data integration

#### Real data (NeurIPS dataset)

To demonstrate the insensitivity of silhouette-based batch removal metrics to differing levels of nested batch effects, we aimed to obtain integration results with varying success. The data were first normalized to median total counts, logarithmized and then dimensionality-reduced with principal component analysis (PCA). No integration (‘none’) served as a baseline. A naive, mild batch correction (‘suboptimal’) was achieved through batch-aware selection of highly variable genes (HVGs), prioritizing genes that were highly variable across batches, which was applied before PCA (carried out with scanpy^22^ (version 1.10.2)). To obtain different batch removal strengths, we used our tunable model liam^14^, which gave us control over distinct batch removal strengths. In particular, we applied liam (version 0.1.1) to the raw scRNA-seq data of the BMMC Multiome dataset with default parameters (‘effective’). Additionally, we increased batch removal by setting the adversarial scaling parameter to 5 (‘optimized’). Note that the findings related to the metrics are not specific to the integration models used.

#### Real data (HLCA and HBCA)

For the HLCA and HBCA datasets, we applied similar integration strategies as described for the NeurIPS dataset, except that we relied on author-provided integrated embeddings for ‘effective’ and had no ‘optimized’ integration. For HLCA, we used the scANVI embedding (key: ‘X_scanvi_emb’); for HBCA, we used the integrated scRNA-seq embedding (key: ‘X_pca’) (effective). For HLCA and HBCA, we applied PCA to the provided normalized counts for no integration (‘none’). For both datasets, the ‘suboptimal’ integration involved batch-aware HVG selection before PCA, considering multiple batch variables for HLCA (‘dataset’, ‘donor_id’ and ‘assay’) and ‘donor_id’ for HBCA.

#### Simulated 2D data and scRNA-seq data (Extended Data only)

No integration was performed as we simulated differing levels of nested batch effects, which could, in turn, be interpreted as varying levels of success at batch effect removal.

### Evaluation

#### Literature review for metric usage

To assess the adoption of silhouette-based metrics for evaluating batch effect removal in single-cell studies, we conducted a systematic literature review. We performed a comprehensive keyword search through the Nature advanced search interface (2010–present) using the following keyword combinations:

- ‘batch silhouette’ and ‘single-cell’
- ‘silhouette batch’ and ‘single-cell’
- ‘ASW batch’ and ‘single-cell’
- ‘batch ASW’ and ‘single-cell’
- ‘bASW’ and ‘single-cell’
- ‘batch effect’ and ‘single-cell’ and ‘silhouette’

We then manually reviewed these papers to identify studies that used metrics adapting silhouette to score batch integration success (for example, batch ASW (cell type) or batch ASW (global)). All papers that we found to use such metrics are cataloged in Supplementary Table 1. The search was last updated on April 10, 2025.

#### Metric overview

We assessed horizontal data integration using a broad selection of metrics, in particular, batch ASW, iLISI, CiLISI, BRAS and BRAS variants for batch removal and cLISI, cell type ASW, NMI cluster/label and ARI cluster/label for bio-conservation.

For the simulated scRNA-seq and NeurIPS data, we used the scib implementations^11^ for these metrics (version 1.1.5), except for the implementations for the custom CiLISI and proposed BRAS metrics (detailed below).

For the HLCA and HBCA data, we used the scib-metrics (version 0.5.5) implementations for these metrics, including our proposed BRAS metrics that we make available as part of this package, except for iLISI, for which we used the scib implementation, and our custom CiLISI implementation (detailed below).

All metrics were scaled to range between 0 and 1, with 1 being optimal. For the silhouette-based metric cell type ASW, this implies that original silhouette scores around 0 correspond to transformed scores of approximately 0.5. We used low-dimensional embeddings as input: PCA embeddings for simulated data, PCA or liam embeddings for the NeurIPS data and PCA or author-provided integrated embeddings for HLCA and HBCA.

#### Custom implementations of batch removal metrics robust to nested batch effects

For CiLISI, we implemented a custom version of CiLISI^19^, a cell-type-adjusted version of iLISI. First, we computed iLISI (range 0–1, scib implementation (version 1.1.5)) per given cell type label, which was summarized into a weighted mean (weighted by the number of cells per cell type label).

To account for nested batch effects in single-cell data, we introduced BRAS, modifying the silhouette score ${s}_{i}$ as described in Eq. (1). Specifically, we redefined ${b}_{i}$ as the mean distance between a cell *i* and all other cells in any other cluster (default in BRAS). We also tested a version with ${b}_{i}$ as the distance between a cell *i* and all other cells in the farthest other cluster (Extended Data Figs. 3b–5b and 6).

The modified silhouette score is computed per cell *i* assigned to a cluster ${C}_{k}$. Following Luecken et al.’s^11^ implementation, we first computed ${s}_{i}$ with the modification described above.

Then, for each cell type label *j* with $|{C}_{j}|$ cells, we define the BRAS score as follows:

$${\rm{BRAS}}_{j}=\frac{1}{|{C}_{j}|}\sum _{i\epsilon {C}_{j}}1-{|s}_{i}|$$

For the final BRAS score, we average over the set of unique cell labels *M*.

$${\rm{BRAS}}=\frac{1}{|M|}\sum _{j\epsilon M}{\rm{BRAS}}_{j}$$

We use cosine distance as the default for BRAS, finding that it provides higher discriminative power than Euclidean distance (Extended Data Figs. 3b–5b and 6).We also compute batch ASW and cell type ASW with cosine distance.

#### Details on ARI and NMI cluster or label

Following Luecken et al.^11^, we optimized (Leiden) clustering with respect to the ARI and NMI metrics across a range of clustering resolutions and show these results in Fig. 2 and Extended Data Figs. 3–6 (Leiden is now the current default in scib; in the original publication, the Louvain algorithm was used). Results in Fig. 2 and Extended Data Figs. 3–6 were derived with clustering resolutions 0–2 and step of 0.1 for the NeurIPS datasets (default scib) and clustering resolutions 0–2 and step of 0.2 for the HLCA and HBCA datasets (default scib-metrics). Results in Supplementary Figs. 1–4 were derived with clustering resolutions 0–2 and step of 0.1 for all datasets. A discussion on the potential limitations of this strategy, its impact on our results and alternative strategies is presented in Supplementary Note 3 and Supplementary Figs. 1–4.

### Reporting summary

Further information on research design is available in the Nature Portfolio Reporting Summary linked to this article.

## Online content

Any methods, additional references, Nature Portfolio reporting summaries, source data, extended data, supplementary information, acknowledgements, peer review information; details of author contributions and competing interests; and statements of data and code availability are available at 10.1038/s41587-025-02743-4.

## Supplementary information

Supplementary Information Supplementary Notes 1–4 and Figs. 1–4.

Reporting Summary

Supplementary Table 1 List of publications from Nature Portfolio Journals using silhouette-based batch removal metrics.

## Data Availability

All data used in this study are publicly available. For details on data processing and usage, please refer to the Methods. NeurIPS data are available from the GEO (GSE194122). HLCA data (https://datasets.cellxgene.cziscience.com/b351804c-293e-4aeb-9c4c-043db67f4540.h5ad) and HBCA data (https://datasets.cellxgene.cziscience.com/63a485bc-cac7-49d2-83ed-8e07ca4efa2a.h5ad) are available from CellxGene. To facilitate reproducibility, the simulated data generated in this study are available on Zenodo (10.5281/zenodo.15642298)^23^.

## Code availability

The scripts and notebooks for data preprocessing, analyses and figure generation are publicly available on Zenodo (10.5281/zenodo.15642298)^23^. Additionally, we added the BRAS metric to the scib-metrics package as of version 0.5.5 (https://github.com/yoseflab/scib-metrics).
