# Supplementary material for: Shortcomings of silhouette in single-cell integration benchmarking
Source: Nat Biotechnol. 2025 Jul 30;44(6):954–8. doi: 10.1038/s41587-025-02743-4 (PMC13271888; doi:10.1038/s41587-025-02743-4)
Supplement: Supplementary file 1 — Supplementary Notes 1–4 and Figs. 1–4. [file 41587_2025_2743_MOESM1_ESM.pdf]

nature biotechnology

Supplementary information https://doi.org/10.1038/s41587-025-02743-4

Shortcomings of silhouette in single-cell integration benchmarking

In the format provided by the authors and unedited

Table of Contents

Supplementary Note 1 .... *3*
Integration evaluation metric overview 3

Supplementary Note 2 .... *3*
Limitations and challenges of integration evaluation 3

Supplementary Note 3 .... *4*
Impact of clustering strategy on bio-conservation metrics ARI and NMI 4

Supplementary Note 4 .... *6*
Impact of identified silhouette limitations on BRAS 6

Supplementary Figures *7*

Supplementary References *11*

Supplementary Note 1

Integration evaluation metric overview

This overview describes all metrics employed in our study and key complementary approaches.

Batch removal metrics

Among alternatives to silhouette for scoring batch removal, some metrics evaluate local batch mixing. While iLISI quantifies batch diversity in local neighborhoods (Korsunsky et al., 2019), kBET uses a statistical test to compare local and global batch label distributions (Büttner et al., 2019). However, they assume uniformity of batches or, in the case of kBET, equal distribution of local and global batch labels. This assumption does not hold if cell types are not shared across all data sets or, more generally, if there is cell type imbalance between data sets. To address this limitation, variants like CiLISI (Andreatta et al., 2024) and a cell type-adjusted kBET implementation in scib (Luecken et al., 2022) stratify the analysis by cell type annotation labels. The clustering metrics NMI and ARI have also been adapted for batch removal evaluation (Tran et al., 2020, Maan et al., 2024), with Maan et al. (2024) including variants accounting for cell type imbalance.

Bio-conservation metrics

Beyond silhouette repurposed as an external clustering metric (Cell type ASW), classical clustering metrics such as ARI and NMI evaluate cell type label clustering and hence discrete cell type label preservation. Supplementary Note 3 discusses how clustering strategies influence ARI/NMI scores. The cLISI metric assesses local neighborhood purity (Korsunsky et al., 2019), while Maan et al. (2024) evaluate integration on the cell type level using a KNN classifier. Isolated label scores determine whether integration retains rare cell types as separate clusters (Luecken et al., 2022). All these metrics are inherently constrained by the quality and granularity of cell type annotations. Additionally, discrete annotations insufficiently capture biological phenomena like trajectories or gradual responses to stimuli. To address these biological complexities, methods like HVG overlap, trajectory conservation, and cell-cycle conservation (variance preservation) can be useful for data sets for which the preservation of such effects is desired (Luecken et al., 2022).

Supplementary Note 2

Limitations and challenges of integration evaluation

Integration metrics should be seen as proxies for performance rather than definitive measures. There is no one-size-fits-all metric suite; instead, metric selection must match the experimental design and biological context, necessitating multiple and complementary metrics. This is especially true when integrating biologically heterogeneous samples aiming to remove technical variation but to preserve biological heterogeneity. Examples include integrating samples stemming from only partially overlapping tissue sections or biologically distinct conditions, such as healthy and disease samples. Similarly, batch effect removal should be understood as batch

effect adjustment, an approximate correction with inherent limitations. These approximate corrections risk introducing new artifacts, especially when prioritizing removing technical variation in cases where technical batches align with meaningful biological signals. However, careful experimental design can help mitigate such issues, for instance, by including replicates.

To keep things simple, our analysis focused solely on healthy samples, regarding inter-donor variation as negligible noise. However, neglecting inter-donor variation is not universally appropriate, especially in studies related to aging or human diversity. If biological donor-level variation is of interest, careful experimental design becomes essential. In the NeurIPS 2021 data set (Luecken et al., 2021), biological donor-level variation is confounded by technical sample-level variation, which is further nested within site-level technical variation. However, the data set also provides same-donor replicates measured at distinct sites, which presents a rare opportunity for a more nuanced integration evaluation. If such information is available, technical batch effects can be assessed specifically within the replicate same-donor subset, as demonstrated in our prior work (Rautenstrauch et al., 2024), not penalizing possible inter-donor-level variation between samples of other donors.

When integrating heterogeneous data (e.g., combining healthy and diseased samples), we generally do not assume that all samples will mix uniformly, and doing so could favor methods that remove meaningful condition-related variation. Again, targeted evaluation of high-confidence subsets, such as technical replicates, can provide a performance proxy. Since replicates are often unavailable, metrics could also be computed separately for distinct biological conditions, evaluating only healthy samples or only diseased samples. Such an approach prevents the inappropriate penalization of biologically meaningful variation.

While we strongly advocate considering multiple metrics for a comprehensive evaluation, care should be taken when aggregating results across metrics into summary scores. Aggregation can obscure important discrepancies between metrics, especially when their scales and sensitivities differ significantly. For example, simple averaging or rank-based aggregation can disproportionately weigh certain metrics, potentially masking poor performance in others. When results are reported only as a single aggregated score, inconsistencies or failures in individual metrics may go unnoticed and are not traceable.

Supplementary Note 3

Impact of clustering strategy on bio-conservation metrics ARI and NMI

To compute ARI or NMI, used here to score bio-conservation, we compare a clustering for any given input to ground truth labels (cell type labels). The choice of clustering algorithm and hyperparameters affects results. Luecken et al. (2022) opted to optimize clustering for the Louvain algorithm with respect to the NMI and ARI metrics across a range of clustering resolutions. This strategy can lead to optimal cluster numbers strongly deviating from the number of ground truth cell types, complicating comparisons and potentially favoring unrealistic solutions. Recently, Maan et al. (2024) chose to optimize Leiden clustering based on the actual number of ground

truth clusters (cell types). The scib-metrics package, an accelerated, Python-only reimplementation of scib metrics from Luecken et al. (2022) (https://scib-metrics.readthedocs.io/en/stable/generated/scib_metrics.nmi_ari_cluster_labels_kmeans.html; last accessed: April 14th, 2025), defaults to k-means clustering for ARI and NMI, with k set to the number of ground truth cell type labels.

A recent study proved that the NMI metric can exhibit biased behavior when the number of detected clusters exceeds the true number of clusters (Mahmoudi & Jemielniak, 2024). In light of this, we sought to assess the impact of different clustering strategies on ARI and NMI results. We compare the results of choosing the maximum score in the full range of tested Leiden resolutions (0-2, step 0.1; full-range max) with choosing a maximum among a limited number of clusters within ±20% of the ground truth cell types (bounded max) with determining the score based on clustering obtained with k-means clustering (kmeans). Supplementary Figures 1-4(a) show at which resolution and respective number of clusters maximal scores were reached, in the full range and in the bounded region. Supplementary Figures 1-4(b) illustrate how choosing the clustering impacts the overall ranking of distinct scenarios for the different data sets. Minor differences may occur between the ARI and NMI full-range scores computed as part of this supplementary analysis, as compared to the results presented in Figure 2 and Extended Data Figures 3-6, due to differences in initialization and clustering resolution range.

The clustering choice impacts results. For example, for the full NeurIPS data scenario "Suboptimal", the "full-range" maximum ARI and NMI score corresponds to a clustering output of 12 clusters, which strongly deviates from the ground truth of 22 clusters (Supplementary Figure 4(a)). In multiple cases, different clustering strategies led to inconsistent rankings, such as for ARI in the NeurIPS data minimal example (Supplementary Figure 1(b)) or NMI in the NeurIPS full data (Supplementary Figure 4(b)). Our fine-grained analysis also highlights that the clustering resolution range considered might need to be adapted to distinct data sets, e.g., for the HBCA data, the chosen range barely includes the real number of clusters (Supplementary Figure 3(a)). Across the four distinct data (sub)sets analyzed, the "bounded max" strategy was the only approach that consistently yielded rankings aligning with expectations. Other strategies occasionally failed, as seen for "kmeans" for ARI and NMI with the HLCA data (Supplementary Figure 2(b)) and "bounded-max" for NMI with the NeurIPS full data. In line with Mahmoudi & Jemielniak (2024), we find that ARI decreases more sharply than NMI when the number of detected clusters exceeds the true number (a behavior typically desired for the practitioner). Additionally, across multiple data sets, the relative difference between scores obtained for different clustering scenarios was higher for ARI than for NMI.

These findings do not affect the main conclusions of our paper regarding silhouette-based metrics but additionally underscore the impact of clustering strategies on evaluation results. While these results are inevitably influenced by biological complexity, including challenges posed by continuous trajectories or highly similar cell types and the quality of ground truth annotations, the consistent performance of the "bounded max" strategy across diverse data sets and the ARI and NMI metric is particularly notable. This robustness suggests it warrants further consideration as a default clustering approach.

Supplementary Note 4

Impact of identified silhouette limitations on BRAS

The proposed batch removal adapted silhouette (BRAS) metric addresses silhouette's nearest-cluster issue identified in batch correction contexts. Notably, BRAS's focus on cluster overlap scoring renders challenges associated with across method output comparison less problematic. Increased separation between clusters inherently yields poorer BRAS scores, aligning with the metric's intended behavior for integration assessment.

However, BRAS remains subject to broader constraints associated with cluster geometry. Here, we postulate that these constraints are less impactful in batch removal contexts when conducting cell type-level evaluations, assuming that cell type labels for any given data set (batch) originate from coherent clusters in their original embedding space. When assigning batch-specific cluster labels per cell type, these groups should maintain spatial cohesion in unintegrated embeddings.

BRAS retrieved correct rankings across all our analyses, including diverse data sets and integration methods. However, suboptimal integration could theoretically distort these originally coherent batch-specific cell type clusters into irregular or fragmented geometries. Additionally, coherence in cell type labels may be lacking for, e.g., coarse-grained annotations or discretized annotations of continuous processes. Therefore, we emphasize that employing complementary quality metrics remains essential for detecting potential integration artifacts that might influence BRAS-based assessments.

Supplementary Figures

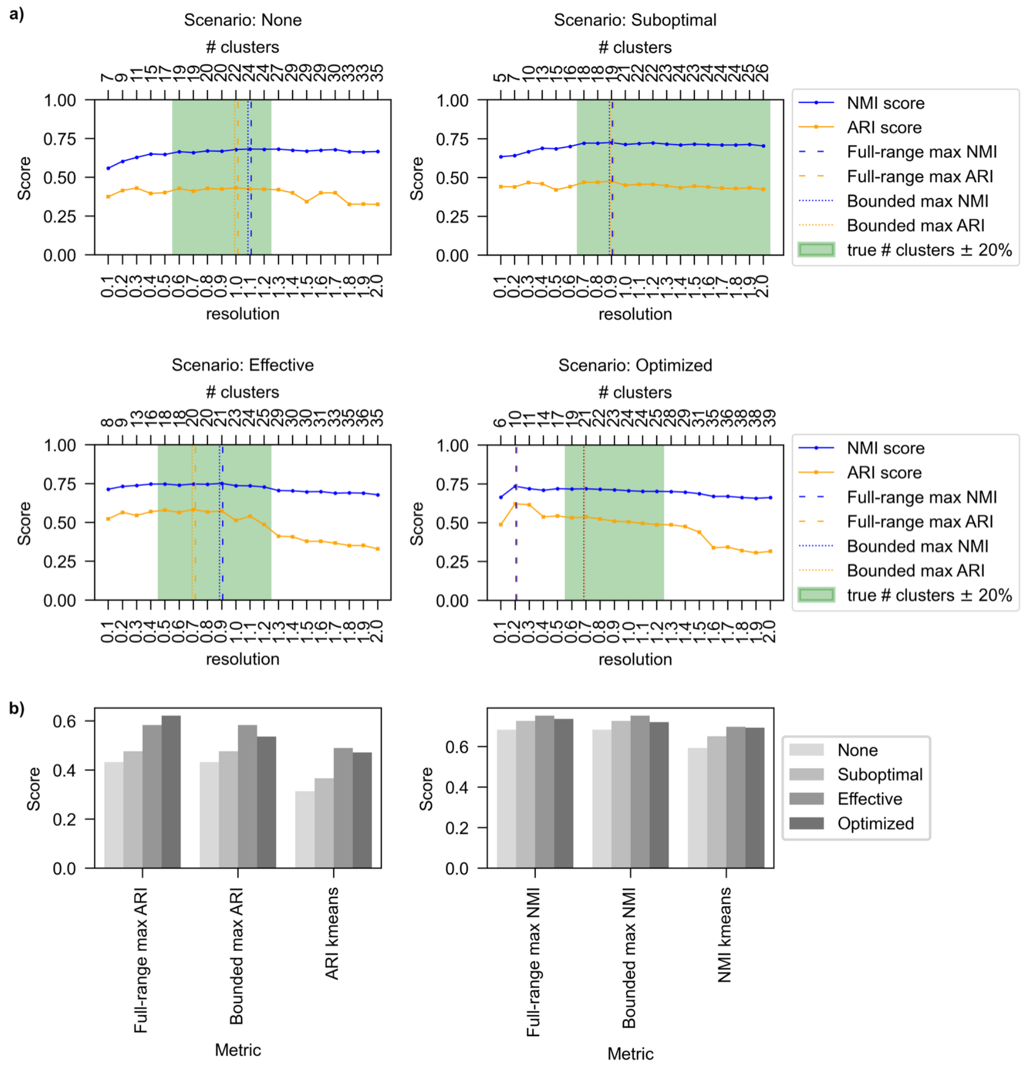

Supplementary Figure 1: Impact of clustering strategy on ARI and NMI bio-conservation metrics for NeurIPS data minimal example. (a) Relationship between Leiden clustering resolution (bottom x-axis), resulting cluster count (top x-axis), and corresponding ARI and NMI scores. Dashed lines indicate resolution and cluster count for maximum metric score across full range (0-2, step 0.1). Green area highlights results within ±20% of true cluster count. Dotted lines show resolution and cluster count for maximum score within bounded range. True cluster count: 22. **(b)** Comparison of max scores from different clustering strategies shown in (a) and clustering obtained with k-means.

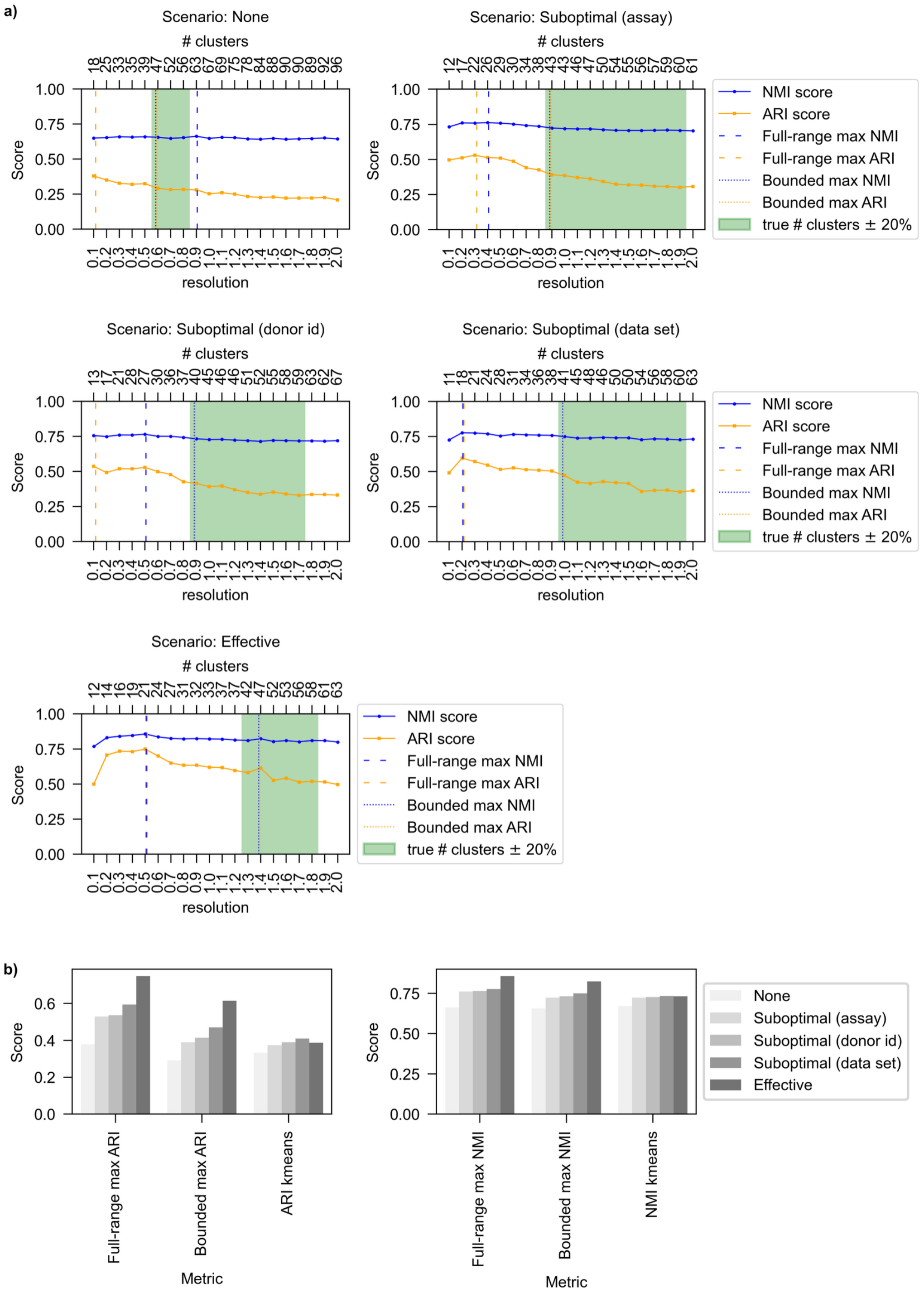

Supplementary Figure 2: Impact of clustering strategy on ARI and NMI bio-conservation metrics for full HLCA data. (a) Relationship between Leiden clustering resolution (bottom x-axis), resulting cluster count (top x-axis), and corresponding ARI and NMI scores. Dashed lines indicate resolution and cluster count for maximum metric score across full range (0-2, step 0.1). Green area highlights results within ±20% of true cluster count. Dotted lines show resolution and cluster count for maximum score within bounded range. True cluster count: 50. **(b)** Comparison

of max scores from different clustering strategies shown in (a) and clustering obtained with k-means.

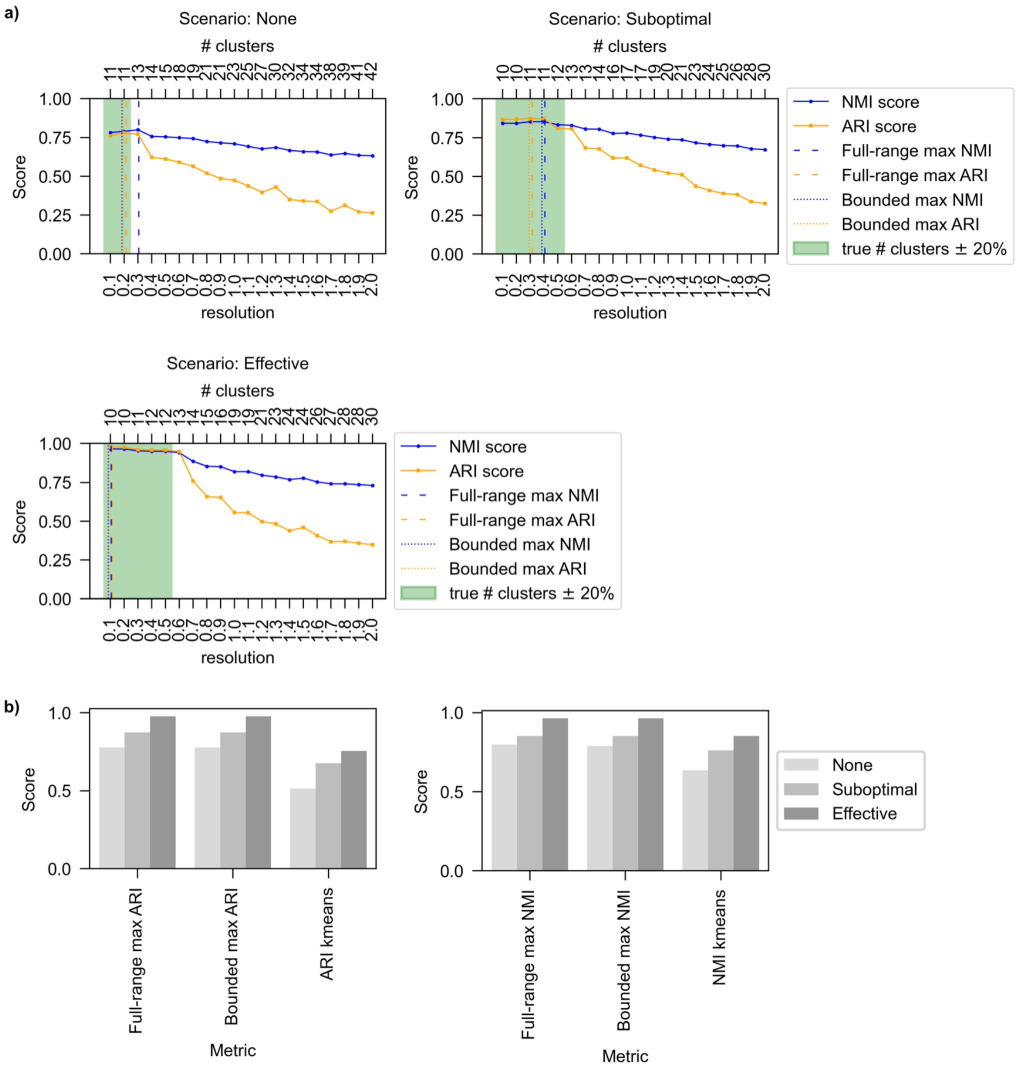

Supplementary Figure 3: Impact of clustering strategy on ARI and NMI bio-conservation metrics for full HBCA data. (a) Relationship between Leiden clustering resolution (bottom x-axis), resulting cluster count (top x-axis), and corresponding ARI and NMI scores. Dashed lines indicate resolution and cluster count for maximum metric score across full range (0-2, step 0.1). Green area highlights results within ±20% of true cluster count. Dotted lines show resolution and cluster count for maximum score within bounded range. True cluster count: 10. **(b)** Comparison of max scores from different clustering strategies shown in (a) and clustering obtained with k-means.

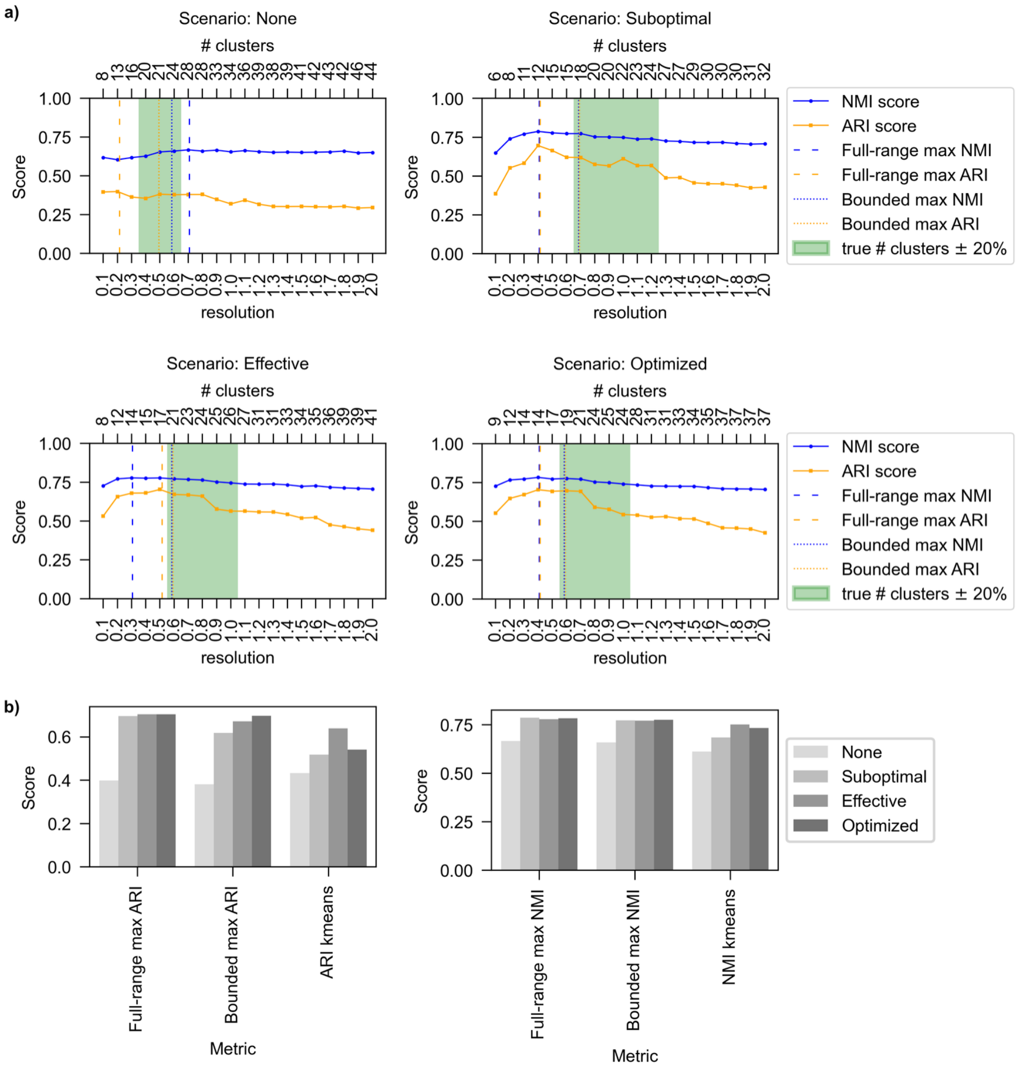

Supplementary Figure 4: Impact of clustering strategy on ARI and NMI bio-conservation metrics for full NeurIPS data. (a) Relationship between Leiden clustering resolution (bottom x-axis), resulting cluster count (top x-axis), and corresponding ARI and NMI scores. Dashed lines indicate resolution and cluster count for maximum metric score across full range (0-2, step 0.1). Green area highlights results within ±20% of true cluster count. Dotted lines show resolution and cluster count for maximum score within bounded range. True cluster count: 22. **(b)** Comparison of max scores from different clustering strategies shown in (a) and clustering obtained with k-means.

Supplementary References

Andreatta, M., Hérault, L., Gueguen, P., Gfeller, D., Berenstein, A. J., & Carmona, S. J. (2024). Semi-supervised integration of single-cell transcriptomics data. *Nature Communications*, *15*(1), 872. https://doi.org/10.1038/s41467-024-45240-z

Büttner, M., Miao, Z., Wolf, F. A., Teichmann, S. A., & Theis, F. J. (2019). A test metric for assessing single-cell RNA-seq batch correction. *Nature Methods*, *16*(1), 43–49. https://doi.org/10.1038/s41592-018-0254-1

Korsunsky, I., Millard, N., Fan, J., Slowikowski, K., Zhang, F., Wei, K., Baglaenko, Y., Brenner, M., Loh, P., & Raychaudhuri, S. (2019). Fast, sensitive and accurate integration of single-cell data with Harmony. *Nature Methods*, *16*(12), 1289–1296. https://doi.org/10.1038/s41592-019-0619-0

Luecken, M. D., Burkhardt, D. B., Cannoodt, R., Lance, C., Agrawal, A., Aliee, H., Chen, A. T., Deconinck, L., Detweiler, A. M., Granados, A., Huynh, S., Isacco, L., Joon Kim, Y., Klein, D., de Kumar, B., Kuppasani, S., Lickert, H., McGeever, A., Mekonen, H., … Bloom, J. M. (2021). A sandbox for prediction and integration of DNA, RNA, and proteins in single cells. *Thirty-Fifth Conference on Neural Information Processing Systems Datasets and Benchmarks Track (Round 2)*. https://openreview.net/forum?id=gN35BGa1Rt

Luecken, M. D., Büttner, M., Chaichoompu, K., Danese, A., Interlandi, M., Mueller, M. F., Strobl, D. C., Zappia, L., Dugas, M., Colomé-Tatché, M., & Theis, F. J. (2022). Benchmarking atlas-level data integration in single-cell genomics. *Nature Methods*, *19*(1), 41–50. https://doi.org/10.1038/s41592-021-01336-8

Maan, H., Zhang, L., Yu, C., Geuenich, M. J., Campbell, K. R., & Wang, B. (2024). Characterizing the impacts of dataset imbalance on single-cell data integration. *Nature Biotechnology, 42*(12), 1899–1908. https://doi.org/10.1038/s41587-023-02097-9

Mahmoudi, A., & Jemielniak, D. (2024). Proof of biased behavior of Normalized Mutual Information. *Scientific Reports*, *14*(1), 9021. https://doi.org/10.1038/s41598-024-59073-9

Rautenstrauch, P., & Ohler, U. (2024). Liam tackles complex multimodal single-cell data integration challenges. *Nucleic Acids Research*, *52*(12), e52. https://doi.org/10.1093/nar/gkae409

Tran, H. T. N., Ang, K. S., Chevrier, M., Zhang, X., Lee, N. Y. S., Goh, M., & Chen, J. (2020). A benchmark of batch-effect correction methods for single-cell RNA sequencing data. *Genome Biology, 21*(1), 12. https://doi.org/10.1186/s13059-019-1850-9
